# Supplementary material for: Characterization of a Family of Scorpion Toxins Modulating Ca2+-Activated Cl− Current in Vascular Myocytes
Source: Toxins (Basel). 2022 Nov 10;14(11):780. doi: 10.3390/toxins14110780 (PMC9699600; doi:10.3390/toxins14110780)
Supplement: Supplementary file 1 [file toxins-14-00780-s001.zip › toxins-1776267-supplementary.pdf]

gene abbreviation	amplified sequence	length		efficiency %	R^2
Ano1 (TMEM16A)	ACAACTCCACCATGAGAACCTTCTCCTTGTGGATCTGCTGGCTGATGTCTTTGGGGATATCAGGGATCACCCAGTCCACAAAGTCACTCATGAACATCACCAGGTTCTGGAAGACAATGACGAATGCCAGCCGGGCGGCCAGGA	114	intron_spanning	100	0,9986
Ano2	GGCAAGTTCTCAGTCATCATCAATGCTTTTGTCATTGCTGTCACCTCCGACTTTATCCCCCGCCTTGTATACCAGTACTCCTATAGCCACAACGGGACGCTACATGGATTCGTGAACCA	119	intron_spanning	99	0.9996
Ano3	CAGGAATCAGCATTCCAGTATACCATCCCAGCCAGGCAAAGTATAAGCCAATCTTCTCACCAAAGTACAGCCTGATTAAGTCCAGAGGTTGATGCTTGTACCACATTCC	109	intron_spanning	102	0.9998
Ano4	CCTTCGAGAATTCCATACCAAATTCCTATGTCCTTGGCCCTCGAAGCCAGAGGCCTTCTCCACTGAGTCACAAACTTGTAAGCATCAAGTCGGATTTCAATGATGTTATTCAGTAAGGCC	120	intron_spanning	99	0.9997
Ano6	AAGTGATGAACAACGTTAACAAATTTGGGATTAATAGACTGGTCAGTTCTGGCATCTACAAAGCAGCGTTCCCTCTGCACGACTGCAAATTCAACTATAAGTCGGAAGACC	111	intron_spanning	94	0.9993
Ano7	GCTGTGGTGATTATGTGTCTTGTATCTATCATCCTGTACCGGGCCGTGATGGCTATCATCGTGTCCAAGTCGAACAACGCCTTC	84	exonic	91	0.9916
Ano8	CGAGGACCTGTTATGTCTCTTCTGTCTCCCGGCCGCATCGCCAGAGCCAGTCTCCGGATCTCGGTCTCCATCAGATC	77	exonic	103	0.9976
Ano9	AGTCTCTTCTTGCTGTTCACGACAAAGTTCACAATTCGGATTCTTGTGGTAGCTATGTTGAAGTTCTGCTTGGCTCTGGACCTAGGATCTTCAGGCTTCATGAGGA	106	intron_spanning	96	0.9994
Ano10	GGAACAACTCCAAGTAATCATCAAAGGTCCCCAGGTAAGTGCCCATTTCCTTCTCCAGGAGGACTTGCTCATACAAGGTCGTGTCAACATCCGACTTCAATGC	103	intron_spanning	101	0.9985
Best1	TGCTGATGATTCGAGAGAACCATTCTGTAGAGTCCACGGATGCAATAGTAGAGGAATATGAAGACAAGGAATTCTCCG	78	intron_spanning	96	0.9993
Clcn1	TCCATGTTCTCTGGTGTCTATGAGCAGCCATACTATTACACTGACATCCTGACAGTGGGCTGTGCCGTGGGGGTCGGTTGCTGTTTTGGAACACCACTTGGAG	103	intron_spanning	98	0.9996
Clcn2	CAGGACGGGTGCGGGCGCGGGCTCAGCGAGCCGGGAGCTGAGTCAAGGCCAGAGCCCGAGAAGCAAGAGGAGGCAAGAGGACAGTGCACCGAGATG	66	exonic	99	0.9997
Clcn3	GACTTCCGAAGACGACAATTTGTTAGACGGTGACACAGCAGCTGGAACTCATTATACAATGACAAATGGAGGCAGCATTAACAGCTCCACACACT	95	intron_spanning	100	0.9990
Clcn4	TGGATCATCTTCAGGAACTGGTGCAGGCCTGGAGCGGACTGTGTCACACCAGGGCTTTATGCGATGGTGGGAGCTGCAGCCTGTCTAGGTGGAGTGACTAGGATGACAGTGTCTCTGG	118	intron_spanning	96	0.9994
Clcn5	CTTCTGTAAAAGCGCCTCAACCTTAATGAGCAAATGCGGGCTGCCAAGGTTGGTTCTTCCTTGAGGCAGAAAAAAAGAAAACTGTGAACGAAGGGAACAAGTCATACAATGGTGGTGGAATAGGCTCTTCAAATAGA	107	intron_spanning	101	0.9993
Clcn6	CATGTCGTTGTAGGTGTCATTGGGACAGAAAAATGTCTTGATAGTTGAATTCACATCTTCCGATGTGACCTGGAGCTGGAATGAGCCATTACCCGTTTGACTCGTGGAAGACATCT	116	intron_spanning	102	0.9992
Clcn7	CCTAAGTGACACAGCGTCTAATCACAATGGGTTCCCTGTGGTGGAGGATGTAGGAGACACCCAGCCAGCCAGACTCCAAGGCCTAATCCTGCGTTCCCAGCTCATCGTGCTCCTGAAGCACAAGGTGTTTGTGGAGAGGTC	111	intron-spanning	97	0.9974
Clcnka	TCGGAATCTGGTCTTGTAGATGGAGGTGATGGTCTCCTGTTCACTGTTGAAGACCCCTAGGAGACGGAACATGAAGG	77	intron_spanning	99	0.9987
Clcnkb	CTCCCTGTACAGCCACTTATGTGCTCTGACCACACGTCCAATAGCAAAGTTCATGGCATAGCTGATCAGAGCCATGAGCACCCCGAGAGCCACCAGGAAGTACCAGTCCTCACCCACACGGAACAGCCGCTCTTTCAGCCACTCTAGGCTCCCCTGGATGTTTCTGCGGATT	172	intron_spanning	97	0.9994
Cftr	GATGTTGGCTATTACTGGATCTACTGGAGCAGGAAAGACATCACTCCTGATGCTGATACTGGGAGAACTGGAAGCGTCAGAGGGAATTATTAAGCACAGTGGAAG	105	intron_spanning	99	0.9998
Gadd45a	CTTAAGGCAGGATCCTTCCATTGTGATGAATGTGGGTTCGTCACCAGCACACAGTGTAAGTCCGGGGTCTGCGCAGCGCCCCCGCTCTCAGCGGGGCTCTTGTCGTTCTCCAGTAGCAACAGCTCTGCCAGCCGACC	107	intron_spanning	100	0.9998
Lrrc8a	AAGACCATCGAGGAGATCATCAGCTTCCAGCATCTGCACCGCCTCACCTGCCTTAAGCTGTGGTACAACCAC	72	exonic	97	0.9997
Lrrc8c	GAACTCGGTCACTGGAATCATGTTTCTTATATGTATATGTTACTTCTCTGAACATCTGTGGTCCCAGAATTCTTAGTCATAAGCAGCCAC	90	intron_spanning	91	0.9993
Lrrc8b	CCAAGAGTAGACACTGAAGTTTCTTGCACTGGAACAGCCCGTCTGGTAGCATCTCGATGTTATTGTTGGTCACGGCGAAGTACTGCAGGTTGGTCAGGTACTGG	104	intron_spanning	101	0.9996
Lrrc8d	TGCAGTGTAGCAGAGGATAAAAATGAACTTGGCGGTCTTGATGAGGGTCTGGACCACGTAGAGCTTGTAGATCAAGTCACTGTCTTCCAC	90	exonic	96	0.9997
Lrrc8e	CTGATGAGTTCTCACGAGATTCATGGCTGGGCAGGCAGATGATCTTGTCCTGTGTCACCTGGAGGGTGCACCCGAACACCCCAATCATGAGCATAGCCACAG	102	intron_spanning	97	0.9996
Sema3g	AGACTCAACAACCTCTCTCTCCCACCCGGCTAGGAAAAGGAGTCCAGGACGCCTGATTACCTCTTAGAGAAAATCTCTACCACCTACAGGAA	92	exonic	99	0.9995
Slc12a1	TGGATGGGTGAAAGGTGTGCTGGTGAGGTGCATGCTGAACATCTGGGGAGTCATGCTCTTCATTCGCCTCTCCTGGATTGTAGGAGAAGCGGGAATTGGTCTTGGAGaTCCTTATAATTCTTCTTTCCACCATGGTAACCTCTATCAC	117	intron_spanning	100	0.9999
Slc12a2	TGTAAGATCCGAGTATTCATTGGTGGAAAGATAAACAGAATAGACCATGACCGGAGAGCGATGGCTACTTTACTCAGTAAATTCCGAATAGACTTCTCCGATATCATGGT	110	intron_spanning	94	0.9984
Slc12a4	GCTTGACATGTCGTGTGTGCCCGATGGGTAAAAGATGGCAGCTGGTGGAGCAATGTAGGTCAGCAAGATCTCAATGGCTCCTAGGAT	87	intron_spanning	99	0.9999
Slc12a5	CCGAGCCCGGCGCCGCGCAGCCACCATGCTCAACAACCTGACGGACTGCGAGGACGGCGATGGGGGAGCCAACCCGGGTGACGGCAATCCCAAGGAGAGCAGCCCCTTCATCAACAGCACGGACACGGAGAAGGGGAGAGAGTATGATGGCAGGAACATGG	113	intron_spanning	96	0.9998
Slc12a6	CATGCTATTACCATTCCTACTGAAACAGCACAAGGTTTGGCGAAAATGCAGCATACGGATCTTCACAGTAGCCCAACTAGAAGACAACAGTATCCAGATGA	101	intron_spanning	96	0.9999
Slc12a7	GTGTTCCTGTCGTGAATCAGCTGGGCCTCTCTCTCCCGCTCATTCTTTGACAACTGCATCTGTTTCAGCATCTGTGACCTCTGCTCCATCATTAGCGTCTTCTCATAGGTGA	112	intron_spanning	99	0.9992
Slc26a9	TTCGTTACCTTCCACACTCTCATCCTTGACATGAGTGGAGTCAGCTTTGTGGACCTGATGGGCATCAAAGCTCTAGCCAAGCTAAGCTCCACCTATGAGAAGA	103	intron_spanning	95	0.9994
Tmem206	GAGCTGGTCCAAGTTCAAGGTCCAGGTGTTGTGCCAGGTGTGGACAATGAGTCTGCATCCAG	62	intron_spanning	99	0.9996
Ttyh1	CCTTGACCTCACAGAGAACAGCCTGCCTCTGCCTCCCTAGTACTGAGATTAAAGCATGTATCACCATATCACCAGC	76	exonic	101	0.9990
Ttyh2	GAGAACGTGCCACTCATCGGGAGAGGTTCCCCTCCGCCCACGTACTCTCCCAGCATGAGAGCCACCTACATGTCCGTGGCGGATGAACACCTGAGACACTAC	102	Intron-spanning	96	0.9995
Ttyh3	CACTCTCGTCTTCGCAATGTCCGGCGCCTGCCTTCCCTTATCCACCCAGCTTACTTGGCTCTTCCTGTC	69	exonic	99	0.9999
Hprt1	GTAGATGGCCACAGGACTAGAACGTCTGCTAGTTCTTTACTGGCCACATCAACAGGACTCTTGTAGATTCAACTTGCCG	79	Exonic	98	0.9989
Sdha	CCTGAGCATTGCAGAATCTTGCAGGCCTGGAGATAAAGTTCCTCCGATTAAGGCAAATGCTGGAGAAGAGTCGGTTATGAATCTTGACAAGTTGAGATTTGCTGATGGAAGTGTAAGAACATCAGAGCTGCGCCTAAGCATGCAGAAG	118	Intron-spanning	97	0.998
Ywhaz	GTTGGAAGGCCGGTTAATTTTCCCCTCCTTCTCCCGCTTCTGCTTCGTCTCCTTGGGTATCCGATGTCCACAATGTCAAGTTGTCTCTCAGTAACTGCA	99	Intron-spanning	92	0.9998
